# Influence of anthropometric, ball impact and landing location parameters on serve velocity in elite tennis competition

**DOI:** 10.5114/biolsport.2023.112095

**Published:** 2022-04-21

**Authors:** Ernest Baiget, Francisco Corbi, José L. López

**Affiliations:** 1 National Institute of Physical Education of Catalonia (INEFC), University of Barcelona (UB), Barcelona, Spain; 2 National Institute of Physical Education of Catalonia (INEFC), University of Lleida (UdL), Lleida, Spain

**Keywords:** Body mass, Body height, Impact height, Impact projection angle, Tennis serve

## Abstract

This study aimed (i) to analyse the associations between serve velocity (SV) and anthropometric, ball impact and landing location parameters in total serves (TS) and fastest serves (FS) performed during an ATP Tour event; (ii) to observe differences between first (S1) and second (S2) serves, and (iii) to determine a SV prediction model based on the relationship between the observed variables. Using Foxtenn technology, 30 S1 and 15 S2 were registered in 14 matches in twenty-one male professional tennis players. Ball impact (impact height [IH], impact projection angle [IPA] and relative impact height [RIH]), bounce landing (width and depth) location parameters, S1 and S2 SV in TS (TSV1 and TSV2) and FS (FSV1 and FSV2) alongside anthropometric characteristics of tennis players (body height [BH], body mass [BM] and body mass index [BMI]) were analysed. Significant moderate to large associations were found between BH and BM and TSV1, FSV1 and FSV2 (r = 0.315 to 0.593; p < 0.001), and between IH and IPA and TSV1 and TSV2 (r = 0.294 to -0.409; p < 0.001). BH and BM were the unique significant contributors of FS explaining 22 to 35% of FSV1 and FSV2. Only BM appears in the model to predict FSV1 and FSV2 (r^2^ = 0.48 and 0.21). We concluded that all three anthropometric, ball impact and bounce landing location parameters small to moderately influence TSV. Anthropometric parameters show an impact on SV when tennis players serve at or near maximal speed, highlighting the influence of BM above BH.

## INTRODUCTION

Tennis serve (S) is a multifactorial nature stroke influenced by several neuromuscular, anthropometric, biomechanical, technical and tactical parameters. From a technical and biomechanical perspective, S has been considered as the most complex tennis stroke [1]. A broad consensus exists in considering that to apply an optimal force production, it is necessary to activate and coordinate multiple segments in the whole kinetic chain including lower limbs, trunk and upper limbs [2]. Specifically, it is necessary to add the forces from the ground up through the kinetic chain, to the upper limbs and finally to the racket [3], being necessary the use of elastic energy and muscle preload [4].

From a tactical and strategic perspective, a high S velocity (SV) and accuracy are a key factor in world-class modern tennis [5, 6]. SV has been considered the greatest contributor to S performance [7], and there is a significant relationship between SV and the probability of winning the point [6]. Nowadays, it is usual to see several SV up to 200 km · h^-1^, and although the Association of Professional Tennis Players (ATP) does not officially recognize SV records, a 263 km · h^-1^ S has previously been registered in a men’s official tennis match. In 2020, the top 10 ATP tennis players won 78.1 ± 7.2% of S games, there were 34 with up to an 80% and 3 players with up to 90% of S games won (https://www.atptour.com/en). The proportion of first S (S1) points won was significantly correlated with competition performance on all surfaces [8]. SV and accuracy determine decisive strategic aspects such as the percentage of points won on S, the number of S games won, or the number of points won directly or with short rallies (i.e., < 4 shots) [8]. This is especially relevant in males who get twice more aces per S game, win 14% more points on S1 and achieve 20% more unreturned S1 than female players [9]. Despite this, tennis performance is characterized by a complex interaction between physical, psychological, tactical, and technical abilities. When analyzing world-class athletes, different player profiles can be observed. Therefore, on which of the aforementioned aspects participants rely on more specifically to develop their game may vary significantly between individuals.

Given the importance of SV on success in professional male tennis competition, the recognition of factors that influence SV is currently relevant for researchers and coaches and therefore it has been widely explored. The influence of different nature parameters of tennis players SV such as anthropometric characteristics [5, 10–12], competitive level [13], isometric [5, 14, 15] and dynamic [5, 10, 15–19] strength, rate of force development (RFD) [15], muscle stiffness [13], isokinetic speed [19], range of motion (ROM) [4, 16], lower and upper limbs joint kinematics and muscle activity [2, 11, 20–24], the match situation [25] or the resistance training [26] have been measured. However most of these investigations were conducted mainly in laboratory/court simulated conditions with absence of a returner [27]. Based on the uncertain nature of the game, tennis players constantly make decisions on ball landing location of their shots [6, 28]. It has been suggested that to appropriately register the S performance the representativeness of S and return is needed [29]. The S without a returner and real match conditions does not allow the server to decide direction, velocity and angle of S depending on the tactical situation.

Although modern technological advances allow to monitor and capture different performance parameters of strokes directly applying video-footage, there are few investigations carried out under real professional tennis events that reflect the influence of parameters related to the ball impact and bounce landing location. Furthermore, the influence of the anthropometric, ball impact and landing location parameters on total (TS) and fastest (FS) S performed during a real match have not been established. To the best of our knowledge, this is the first study that explores the influence of anthropo-metric, ball impact and bounce landing location parameters on first and second (S2) SV in total (TS) and fastest (FS) serves during an ATP competition, comparing differences between S1 and S2. Thus, the aims of the study were (a) to analyse the associations between SV and anthropometric, ball impact and landing location parameters in TSV and FSV in professional tennis players during an ATP Tour event; b) to observe differences between S1 and S2 and (c) to determine a SV prediction model based on the relationship between the observed variables.

## MATERIALS AND METHODS

### Participants

The inclusion criteria for the study were world-class male professional tennis players who participated in the singles main draw of the 2019 Barcelona Open Banc Sabadell and played in the central court of the tournament. The data of twenty-one male professional tennis players (mean ± SD; age, 26.4 ± 5.4 years; height, 186.9 ± 7.4 cm; BM, 81.6 ± 7.1 kg; body mass index [BMI], 23.4 ± 1.1 kg · m^-2^) were used for the performance analysis. The mean ATP ranking of the players was 42.2 ± 37.9 ranging from 2 to 155 and 81% were right-handed. Permission to use the data and the installations was granted by Reial Club de Tennis Barcelona · 1899. The study was performed in accordance with current ethical standards, established in the Declaration of Helsinki of the AMM (2013) and approved by the Clinical Research Ethics Committee of the Catalan Sports Administration (08_2020_CEICGC).

### Experimental Design

The collection of data took place during an official ATP 500 outdoor red clay tournament (22–28 April 2019). The main draw of the tournament included a total of 48 elite tennis players of which 43.8% were analysed (http://www.protennislive.com/posting/2019/425/mds.pdf). For data analysis and in order to match the number of S evaluated in each player, the first 45 S per player (30 S1 and 15 S2) that landed in the service box (no foot fault or net cord) were registered in 14 matches between the second and fourth round, resulting in a total of 945 S (630 S1 and 315 S2) analysed. Data collection approximates to the professional S profile, the proportion of S1 and S2 in elite tennis are around 60 and 40% [8] and competition tennis players hit around 50 and 150 S during a match [30]. Matches were played approximately from 11 am to 20 pm. Parameters related to the ball impact point (impact height [IH], impact projection angle [IPA] and relative impact height [RIH]), ball bounce landing location (width and depth) and S1 and S2 velocities (SV1 and SV2) were registered for players in TS and FS. Moreover, anthropometric characteristics of professional tennis players (body height [BH], body mass [BM] and body mass index [BMI]) were registered. This procedure was used to determine to what extent anthropometric (BH, BM and BMI), ball impact (IH, IPA and RIH) and bounce landing location (depth and width) related to SV1 and SV2. On the other hand, multiple regression analyses were used to develop models that were most effective at predicting SV1 and SV2.

### Measurements

**FIG. 1:**
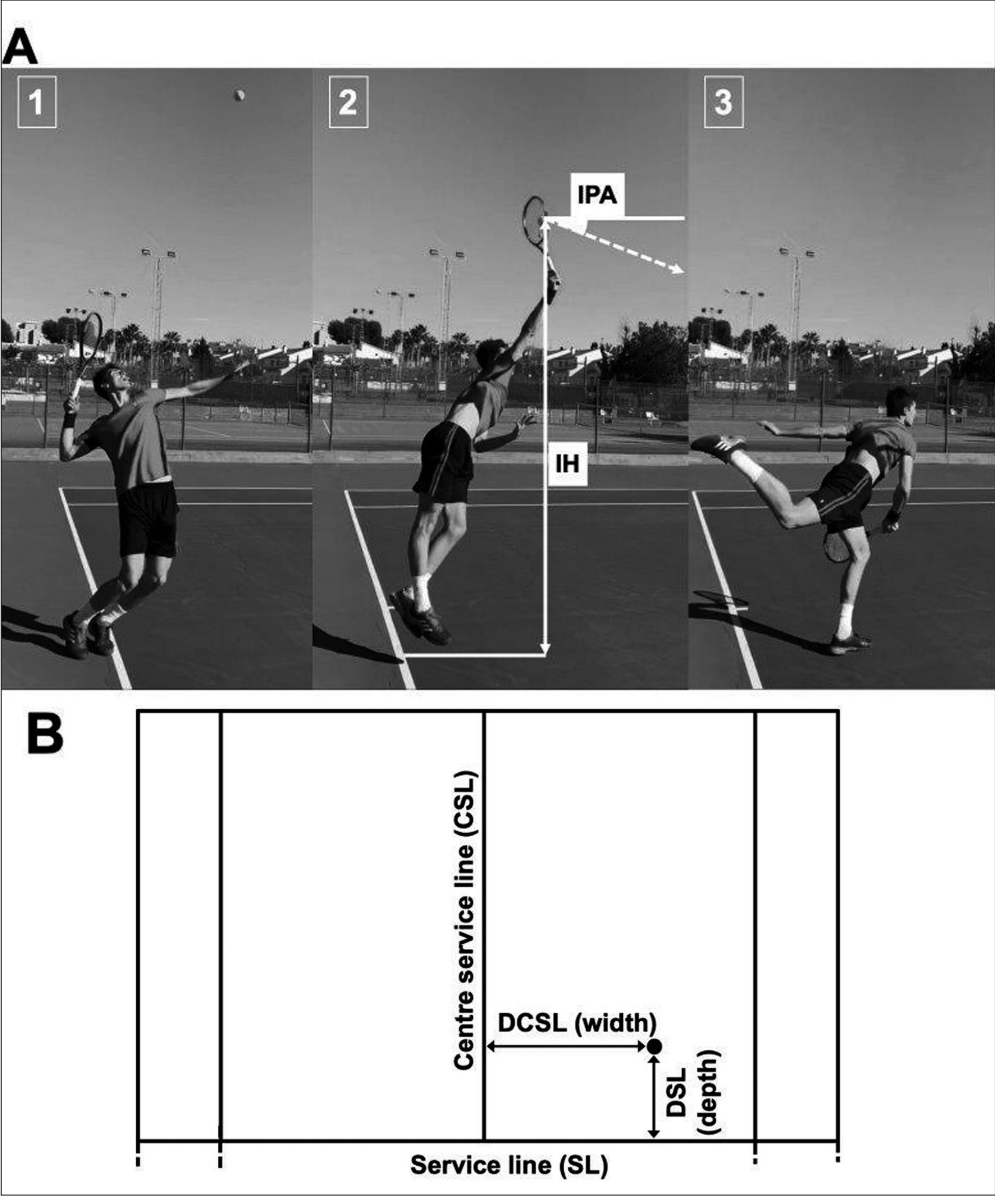
Methods used to calculate ball impact (A) and landing location (B) parameters of tennis serve. Impact angle (IPA), impact height (IH), depth (distance to the service line; DSL) and width (distance to the centre service line, DCSL) are shown. Selected images of preparation phase (1, loading stage), acceleration phase (2, contact stage) and follow-through phase (3, finish stage).

Peak SV and ball impact and landing location parameters were recorded in real time by Foxtenn’s technology (Foxtenn Bgreen, SL, Spain) currently used in ATP, WTA and ITF professional tennis events. Foxtenn’s analysis technology system allows to capture ball trajectories through an automatic tracking of the ball that includes instantaneous ball position and ball velocities are also provided. Ball trajectories tracking is based on capturing images by multiple high-speed cameras of the real ball bounce in combination with high-frequency laser scanners fixed around the court (https://www.itftennis.com/media/6215/pat-16-014-pat-approval-report-foxtenn-diamond-final-v0.pdf). Specifically, the ball impact and bounce location parameters were determined by a high-speed camera (300 fps) positioned in the center of the baseline at a height of 8 m and 10 synchronized laser scanners (100 Hz each one) situated close to the ending lines at ground level and using as reference parameter the centre service line and the service line. Peak SV was determined using two radar guns (Stalker ATS II, United States, frequency: 34.7 GHz (Ka-Band) ± 50 MHz) positioned in the center of the baseline at a height of 2 m. The cameras, laser scanners and radar guns were wired to a server and a designated operator controlled the system via software running on a second server (connected to the first server). The system meets International Tennis Federation (ITF) criteria (https://www.itftennis.com/media/7365/elc-evaluation-paper-revision-26.pdf) of accuracy and reliability (https://www.itftennis.com/media/7203/line-calling.pdf) and is approved by the Women’s Tennis Association (WTA) and ATP. Depth of service landing location was determined by the distance from the bounce to the service line (DSL) and width lateral bounce location by the distance from the centre service line (DCSL) (Figure 1B). IPA was determined by the ball impact projection angle from the horizontal (vertical projection angle) and IH by the distance in cm from the ball at the impact moment to the ground (Figure 1A). Figure 1A shows the three phases of specific serve model [1] and the ball impact parameters (IH and IPA) are located in the acceleration phase (second phase). Anthropo-metric characteristics of professional tennis players (BH and BM) were obtained from publicly available information listed in the official ATP website as in previous studies conducted in elite tennis players [12, 31]. RIH was determined by the relationship between IH and BH: (IH/BH)*100.

### Statistical Analyses

Descriptive data are expressed as mean ± standard deviation (SD) and 95% confidence intervals (95% CI). The normality of variables distribution was assessed with the Shapiro-Wilk test. Most of parameters at TS1 (SV, IH, RIH and DCSL), TS2 (IH, RIH and IPA), FS1 (IPA and DCSL) and FS2 (RIH, IPA and DCSL) did not have a Gaussian distribution, so nonparametric test were used. Friedman’s test was used to discern any significant differences between S1 and S2. Wilcoxon’s test was used to identify those differences. Mean percent differences values were also used. The magnitude of the differences in mean was quantified as effect size (ES) and interpreted according to the criteria used by Cohen [32] < 0.2 = trivial, 0.2–0.4 = small, 0.5–0.7 = moderate, > 0.7 = large. Spearman rank order correlation coefficients for non-parametric data were used to examine the relations between SV and anthropometric, ball impact and bounce variables, while coefficient of determination were used to explain the common variance between these variables and SV. Correlations were classified as trivial (0–0.1), small (0.1–0.3), moderate (0.3–0.5), large (0.5–0.7), very large (0.7–0.9), nearly perfect (0.9), and perfect (1.0) [33]. As DSL, and SV at FS1 and FS2 were normally distributed, parametric statistical analysis were conducted. Paired t-test was used to discern any significant differences between S1 and S2 and Pearson correlation coefficient was used to quantify the relationship between FS1, FS2 and DSL. TSV1, TSV2, FSV1 and FSV2 were used as the dependent variables in the stepwise multiple regression analysis, whereas anthropometric data (BH, BM, BMI) and ball impact variables (IH, IPA, RIH) operated as independent predictors. Ball landing location parameters (width and depth) were not used as independent predictors because it was considered that they depend on SV rather than the opposite. Statistical significance was accepted at an alpha level of p ≤ 0.05. All statistical analyses were performed using IBM SPSS Statistics 26.0 (SPSS, Inc., Chicago, IL.).

## RESULTS

**FIG. 2:**
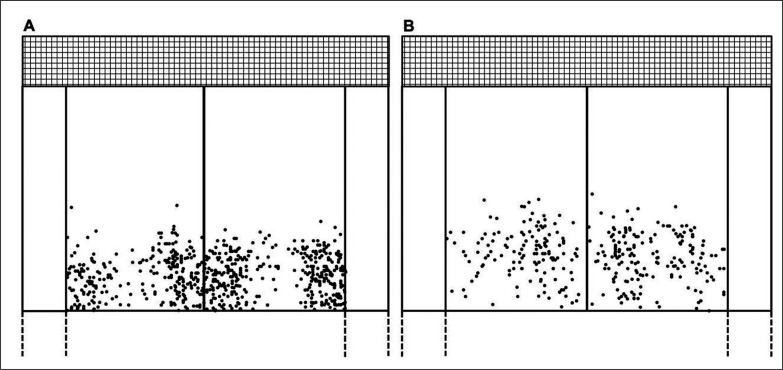
First (A) and second (B) total serves (TS1 and TS2) bounce landing locations.

TS1 and TS2 bounce landing locations are shown in Figure 2. TS2 and FS2 showed a large and a moderate to large reduction of SV (ES = 1.30 and 2.61, -16.7 and -18.6%; p < 0.001) and depth (increase in DSL: ES = -0.63 and -1.08, 53.9 and 53.1%; p < 0.001 and < 0.01) and a small to large increase in IPA (ES = -0.42 and -0.75, 20.1% and 16.5%; p < 0.001 and < 0.01) compared to the TS1 and FS1. No differences (p > 0.05) and small to trivial ES were found between IH, RIH and width at S1 compared to S2 (Table 1).

**TABLE 1:** Serve velocity (SV), impact (IH, IPA and RIH) and landing location (DCSL and DSL) parameters and differences between first and second total (TS1 and TS2) and fastest (FS1 and FS2) serves.

	TS (n = 945)						FS (n = 42)					
	TS1 (n = 630)	TS2 (n = 315)	Difference				FS1 (n = 21)	FS2 (n = 21)	Difference			
			*p*	*ES*	Descriptor	%			*p*	*ES*	Descriptor	%
SV (km · h^-1^)	173.6 ± 18.0 (172.4–175.1)	144.7 ± 13.1* (143.2–146.1)	< 0.001	1.30	Large	-16.7	201.3 ± 9.9 (196.8–205.8)	163.8 ± 9.2* (159.6–168.0)	< 0.001	2.61	Large	-18.6

**Ball impact location parameters**												
IH (cm)	303.4 ± 11.9 (302.4–304.3)	303.3 ± 12.1 (302.0–304.7)	0.416	0.04	Trivial	-0.03	302.1 ± 11.3 (296.9–307.2)	304.3 ± 11.3 (299.1–309.4)	0.297	-0.25	Small	0.73
RIH (%)	162.5 ± 2.2 (162.2–162.6)	162.4 ± 3.0 (162.0–162.7)	0.321	0.04	Trivial	-0.1	161.7 ± 2.3. (160.7–162.7)	163.0 ± 5.5. (160.5–165.5)	0.309	-0.27	Small	0.80
IPA (**°**)	-4.37 ± 1.19 (-4.47– -4.29)	-3.49 ± 1.35* (-3.63– -3.34)	< 0.001	-0.42	Small	20.1	-4.62 ± 0.67 (-4.31– -4.57)	-3.86 ± 0.96* (-3.41– -4.30)	0.003	-0.75	Large	16.5

**Ball landing location parameters**												
Width – DCSL (cm)	219.3 ± 138.0 (208.5–230.1)	209.1 ± 102.9 (197.7–220.5)	0.848	0.01	Trivial	-4.7	202.6 ± 149.0 (134.8–270.4)	155.9 ± 107.6 (106.0–204.9)	0.339	0.25	Small	-33.0
Depth – DSL (cm)	101.6 ± 57.1 (97.2–106.1)	156.4 ± 73.7* (148.3–164.6)	< 0.001	-0.63	Moderate	53.9	70.8 ± 54.8 (45.8–95.8)	146.6 ± 62.5* (118.2–175.0)	< 0.001	-1.08	Large	53.1

Data are mean ± SD (95% confidence interval). TS = total serves performed; TS1 = total first serves performed; TS2 = total second serves performed; FS = fastest serves; FS1 = fastest first serve; FS2 = fastest second serve; SV = serve velocity; IH = impact height; IPA = impact projection angle; RIH: relative impact height; DCSL = distance to the centre service line; DSL = distance to the service line; ES = effect size;. Note: Magnitudes of ESs were assessed using the following criteria: < 0.2 = trivial, 0.2–0.4 = small, 0.5–0.7 = moderate, > 0.7 = large.

* Significantly different from first serve.

The correlation coefficients between the measured variables and TSV and FSV are presented in Table 2. Significant moderate and large positive correlations were found between anthropometric parameters (BH and BM) and TSV1, FSV1 and FSV2. The coefficient of determination between BH and BM and FSV1 and FSV2 ranged from 22 to 35%. Significant moderate positive (IH) and small to moderate negative (IPA, DCSL and DSL) correlations were found between ball impact and bounce landing location and TSV1 and TSV2. No significant correlations were found between ball impact and bounce landing location parameters and FSV.

**TABLE 2:** Correlation coefficients (r) between anthropometric (PH, BM and BMI), ball impact (IH, IPA and RIH) and landing location (DCSL and DSL) parameters and first and second total (TSV1 and TSV2) and fastest (FSV1 and FSV2) serves velocities.

	TSV (n = 945)						FSV (n = 42)					
	TSV1 (km · h^-1^)			TSV2 (km · h^-1^)			FSV1 (km · h^-1^)			FSV2 (km · h^-1^)		
	*r*	*p*	r^2^	*r*	*p*	r^2^	*r*	*p*	r^2^	*r*	*p*	*r*
**Anthropometric parameters**												
BH (cm)	0.318	< 0.001	0.101	0.016	0.772	0.000	0.503	0.020	0.253	0.486	0.025	0.236
BM (kg)	0.315	< 0.001	0.099	0.075	0.186	0.006	0.593	0.005	0.352	0.466	0.033	0.217
BMI (kg · m^-2^)	0.128	0.001	0.016	0.073	0.195	0.005	0.264	0.247	0.070	0.125	0.588	0.016

**Ball impact location parameters**												
IH (cm)	0.294	< 0.001	0.086	0.329	< 0.001	0.108	0.419	0.059	0.176	0.135	0.560	0.016
RIH (%)	-0.062	0.118	0.004	-0.050	0.379	0.003	-0.212	0.357	0.045	-0.397	0.075	0.158
IPA (**°**)	-0.391	< 0.001	0.153	-0.409	< 0.001	0.167	-0.177	0.443	0.031	-0.143	0.536	0.020

**Ball bounce landing location parameters**												
Width-DCSL (cm)	-0.173	< 0.001	0.030	-0.190	0.001	0.036	-0.127	0.585	0.016	0.039	0.867	0.001
Depth-DSL (cm)	-0.169	< 0.001	0.029	-0.179	0.001	0.032	-0.196	0.394	0.038	0.043	0.855	0.002

TSV = total performed serves velocity (all serves included); TSV1 = total first serve performed velocity; TSV2 = total second serves performed velocity; FSV = fastest serve velocity; FSV1 = fastest first serve velocity; FSV2 = fastest second serve velocity; BH = body height; BM = body mass; BMI = body mass index; IH = impact height; IPA = impact projection angle; RIH = relative impact height; DCSL = distance to the centre service line; DSL = distance to the service line; *r* = correlation coefficients; r^2^ = determination coefficient.

Results of the stepwise multiple regression analysis to explore how the anthropometric and ball impact location values predicted the TSV and FSV are summarized in Table 3. BM and IPA analysed together explained 16% of the variation observed in the TSV1 (adj r^2^ = 0.158; p < 0.001) and IPA, IH, BH and RIH explained 20% of the variation in the TSV2 (adj r^2^ = 0.197; p < 0.001). Only the BM appeared in the predictive models of the FSV1 and FSV2 and explained 48 and 21% of the variation respectively (adj r^2^ = 0.480 and 0.208; p < 0.001 and p = 0.022, respectively).

**TABLE 3:** Anthropometric and ball impact variables included in the stepwise multiple regression analysis to explain the variance on first and second total (TSV1 and TSV2) and fastest (FSV1 and FSV2) serves velocities.

Dependent variables	Step	Independent variables entered	Correlations			SEE	*p*
			r	r^2^	Adj. r^2^		
**TSV1 (n = 945)**	1	BM	0.340	0.115	0.114	16.9	< 0.001
	2	BM and IPA	0.401	0.161	0.158	16.2	< 0.001

**TSV2 (n = 315)**	1	IPA	0.390	0.152	0.150	12.1	< 0.001
	2	IPA and IH	0.422	0.178	0.173	12.0	< 0.001
	3	IPA, IH and BH	0.441	0.194	0.187	11.9	< 0.001
	4	IPA, IH, BH and RIH	0.455	0.207	0.197	11.8	< 0.001

**FSV1 (n = 21)**	1	BM	0.712	0.506	0.480	7.1	< 0.001

**FSV2 (n = 21)**	1	BM	0.498	0.248	0.208	8.2	0.022

TSV1 = total first serves velocity; TSV2 = total second serves velocity; FSV1 = fastest first serve velocity; FSV2 = fastest second serve velocity; BM = body mass; IPA = impact projection angle; RIH = relative impact height; Adj. r^2^ = adjusted coefficient of determination; SEE = standard error of estimate.

## DISCUSSION

The main purpose of this study was to analyse the associations between SV and anthropometric, ball impact and landing location parameters in professional tennis players during an ATP Tour event and to observe differences between S1 and S2. Among the variables registered, anthropometric (greater BM and BH), ball impact (higher IH and IPA) and bounce landing (minor width and larger depth) location parameters small to moderately influence TSV. However, the present findings reinforce the influence of anthropometric parameters (BH, BM) in SV when tennis player try to maximise ball velocity (i.e., TSV1, FSV1 and FSV2), but not when S are more conservative (i.e., TSV2). When tennis players serve at maximal SV (FSV1 and FSV2), the anthropometric parameters were the unique significant contributors of SV explaining 22 to 35% of SV. Moreover, the prediction model highlights the importance of BM above BH on FSV. S1 shows a large increase of SV in combination with moderate to large increases in depth and IPA compared to S2.

Regarding BH, it clearly seems that the tallest tennis players have a better disposition to serve fast, as its influence was large in FSV1 (r = 0.503), moderate on TSV1 and FSV2 (r = 0.318 and 0.486) and no influence was found in TSV2. This fact involves that as more powerful a S is, the influence of BH on SV is increased, emphasizing the importance of the principle of force production above the influence of longer connecting body segments. These results are somehow expected in agreement with studies registered in professional tennis tournaments such as the four Grand Slams (r = 0.31–0.57) [31] and Wimbledon (SV1, r = 0.640, p < 0.001) [11]. Greater BH allows a biomechanical benefit to SV over lower BH, as longer limbs allow to obtain higher peripheral head racket velocity at ball impact getting greater hand-racket angular momentum with the equal angular speed of upper body segments [15, 31, 34]. This association seems to be higher when S is tested in laboratory-court simulated conditions in professional tennis players for both S1 (r = 0.78, p < 0.05) and S2 (r = 0.80; p < 0.05) [10]. Possibly, the increase of the effect of BH in closed situations is because there is no decision making and decisive tactical factors of S performance involved (i.e., depth, directions or accuracy). Players can focus only on SV execution, and this fact supports the hypothesis of the relevance to analyze the S influencing parameters during real competition.

In the same way as BH, BM demonstrated large associations with FSV1 (r = 0.593), moderate with TSV1 and FSV2 (r = 0.315 and 0.466) and no associations were found with TSV2. Moreover, BM explained 35% of the variability of FSV1. In line with this, moderate and large associations have been found in junior tennis players (r = 0.44–0.57; r = 0.68) [5, 15], but on the contrary, no associations have been found in S performed in a closed situation (r = -0.22 and -0.15; p > 0.05) [10]. Interestingly, the unique step in FSV1 (adj. r^2^ = 0.48) and FSV2 (adj. r^2^ = 0.21) and the first step in TSV1 (adj. r^2^ = 0.11) included in the multiple regression analysis demonstrate the relevance of BM when players serve at or near maximal SV. The potential biomechanical factors that may cause the clear influence of BM on maximal SV are related to the principle of force (mass × acceleration) and torque production [5, 11]. Based on allometry assumption, BM is associated with torque production and an increased torque would reinforce SV [11]. In this sense, Gale-Wats & Nevill [35] established that the body structure (i.e., body composition) in world-class tennis players has been changing in recent times increasing their muscle mass and experiencing a transformation from endurance to power athletes. The same researchers suggested that a greater muscle mass and the ability to generate power production in all strokes in professional tennis players is associated with greater tennis performance, while taller but less muscled players (i.e., more linear body shape) would perform worse. However, although in professional tennis players we could assume that a high BMI is related to a greater muscle mass, we observed only a small influence of BMI in TSV1 (r = 0.128), but not in FSV. Contrary, in competition but not ATP level tennis players, BMI was largely associated (r = 0.577; p < 0.05) with SV [11] and moderate significant (r = 0.32 to 0.40; p < 0.001) and no significant (r = 0.31, p > 0.05) associations have been found in junior tennis players [5, 15]. Considering that BMI represents an interaction of two parameters (i.e., BM and BH), it is possible that a single increase in BM does not generate a significantly increase in SV and an optimal relationship probably exist between BM and BH to optimize SV.

Regarding ball impact location parameters (IH and IPA), they show a comparable influence to anthropometrics on TSV1 (r = 0.294 and -0.391), but contrary to BH and BM, a higher influence on TSV2 (r = 0.329 and -0.409) and no significant association with FSV. It seems that the benefit of ball impact location parameters on SV was lower when players try to maximize SV, however, we could speculate that they have a major impact on S accuracy. In addition to BH, there were diverse factors that could affect IH, such as vertical jump height, the vertical height of the hitting shoulder, the impact point on the string and the sum of arm and racquet length [7, 10, 31]. Also, it has been argued that IH could affect the precision of S. It has been stated that a higher IH contributes to a larger accessible area in the service box [7] and allows a greater SV with the equal probability of the ball landing inside the service box [31]. RIH was introduced in the analysis as an IH and BH ratio, as it has been found that IH represents the 160% of BH showing the repercussion on IH of the factors that accompany BH (i.e., vertical jump height and vertical height of the hitting shoulder). RIH did not show any influence in SV, thus we could speculate that even if BH has a large influence on SV, the factors that accompany BH to determine IH does not affect SV. No differences in IH and RIH were found between S1 and S2, showing that these parameters are relatively stable in different tennis S (i.e., flat or topspin S). In this same line, no significant differences have been observed between S1 and S2 jump height [17]. It has been stated that in the S there is a higher biomechanical consistency of impact conditions than groundstrokes or volleys as a result of the initially stationary location on the court [27] and that players are able to dominate the main ball impact positions and to reproduce systematically the S performance [36].

Regarding IPA and in the same way that IH, a moderate influence on TSV was found, showing that a large IPA favours TSV but not FSV. However, moderate and large differences have been found between IPA in S1 and S2. In this same line, Chow et al. [37] observed greater IPA in the S1 than in S2 (6.3 ± 1.8° vs 3.1 ± 2.0°). We consider that IPA interpretation should be done alongside the analysis of depth (DSL) and ball velocity. The large differences found between S1 and S2 in depth (˜50%) and ball velocity (˜17–18%) together with the large differences in IPA (20 and 16.5%), shows that the S1 are significantly faster, deeper and with a major IPA than S2. Probably IPA may vary between flat and spin S and the less IPA and depth in S2 can be attributed to the slower SV and greater topspin. In S related with more speed and aggressive action (i.e., power S) players used flat S (i.e., minimum amount of spin on the ball) [22], contrary the S2 are conservative using topspin or slice which reduces SV [6]. A minor spin and higher SV are characterized by straighter ball trajectories over flight than slower S, and consequently, with a reduced clearance over the net to land in the service box. Contrary, on topspin S with greater ball spin rate and less ball velocity (i.e., S2) the Magnus force provokes further curved ball trajectory and lower forward velocity and depth [37, 38]. Therefore, the straight ball trajectory of fastest S needs a large IPA and allows it to reach a greater depth. From a tactical perspective, the greater SV and deeper landing location in S1 compared to S2 leads to a smaller margin of error, however it has been established that a greater SV offers a higher probability to win the S [39]. In agreement with this, the top 10 ATP tennis players until April 2020 won the 71.3 ± 4.0% of points played with the S1 and 49.7 ± 7.2% with S2 (https://www.atptour.com/en).

Visual inspection shows that S1 ball bounces (Figure 2A) are located closer to service centre line (i.e., T location) and singles sideline (wide location) and less into the centre service box (i.e., body location) than S2 (Figure 2B). Alongside a higher SV1 than SV2, the differences between S1 and S2 landing location probably occurs because tennis players try to optimize the tactical advantage, number of aces or points directly won with S to a greater extent in the S1 than S2. In this same line, it has been shown that S1 are more effectively near to T location or near to the singles sideline (wide location) and body serves occur to a lesser extent [40]. Nevertheless, the negative moderate and small associations found between width and TSV1 and TSV2 (r = -0.173 and -0.190) shows a trend that fastest TS were closer to the centre service line (i.e., T location), especially in TSV1. An explanation for this could be that when players seek wide S, they tend to focus on accuracy of S to move the opponent off the court and achieve a positional advantage. Instead, when serving near the T location they seek to maximise S speed and diminish the time reaction of the opponent.

From a practical standpoint, our data emphasizes the influence of selected anthropometric (BM and BH) parameters on SV in professional tennis players during an ATP Tour event and reinforces the BM as an important influencing factor when players serve at or near maximal SV. In this regard, because BH can’t be modified in adult tennis players, it can be postulated that neuromuscular strength training interventions aimed at increasing lean BM can be helpful for improving SV, especially in not very tall professional tennis players (i.e., < 185 cm). However, this assumption should be taken with caution since the ability to accelerate, decelerate and change of direction over short distances is paramount in tennis performance. A greater BM will produce a greater inertia demanding higher force production to produce a given change in velocity or direction [41], so an optimal interaction between increasing BM and COD performance should be found.

There are some limitations that need to be considered when interpreting the results of the present study. First, anthropometric characteristics of tennis players (i.e., BH and BM) were obtained from publicly available information listed in the official ATP website, assuming that the provided data may differ minimally from real data. Second, previously discussed, S is a highly complex stroke determined by different nature parameters [1, 3], however the presented SV prediction model is based on a partial spectrum of serve performance (i.e., some anthropometric [BH and BM], ball impact and bounce variables). The model would be more accurate considering other determinant factors such as technical [39], strategical [6], range of motion [16, 24] or muscle power performance [5, 14–16] parameters.

## CONCLUSIONS

In conclusion, the results of the present study have shown that anthropometric (greater BM and BH), ball impact (higher IH and IPA) and bounce landing (minor width and larger depth) location parameters small to moderately influence SV during an ATP Tour event. However, when tennis players serve at or near maximal SV, the influence of anthropometric parameters are increased being the major contributors to SV. The prediction model constructed highlights the influence of BM above BH on the fastest serves, suggesting its importance to generate power production at the professional tennis level. Moreover, S1 are deeper and show a greater IPA compared to the S2.
